# Prostate Cancer in a Male with Holt-Oram Syndrome: First Clinical Association of the *TBX5* Mutation

**DOI:** 10.1155/2013/405343

**Published:** 2013-08-05

**Authors:** Noel J. Aherne, Guhan Rangaswamy, Pierre Thirion

**Affiliations:** ^1^Department of Radiation Oncology, North Coast Cancer Institute, Coffs Harbour, NSW 2450, Australia; ^2^Department of Radiation Oncology, St. Luke's Hospital, Dublin, Ireland

## Abstract

Holt-Oram syndrome is an autosomal dominant disorder which is caused by mutations of *TBX5* and is characterised by cardiac and skeletal abnormalities. *TBX5* is part of the T-box gene family and is thought to upregulate tumour cell proliferation and metastasis when mutated. We report the first clinical case of prostate cancer in an individual with Holt Oram syndrome.

## 1. Introduction

Prostate cancer is the most common male solid cancer and has a complex aetiology relying on interaction between both genetic and nongenetic factors. While it is known that the existence of one or more hereditary prostate cancer genes is supported through the existence of prostate cancer families, the identification of causative genes for prostate cancer has been elusive to date. While linkage studies on extended high risk families can compare genotypes between affected and nonaffected individuals within these high risk families, it is not currently possible to differentiate between those cases of sporadic versus inherited prostate cancer. One area of study in prostate cancer genetics is the T-box transcription family, which is also responsible for Holt-Oram syndrome. T-box factors are highly conserved transcription factors that act in cell cycle regulation and in the development of malignancy as well as in embryonic development. Their overexpression is thought to downregulate expression of cellular cyclin-dependent kinases which thus allows cells to bypass programmed senescence. This case outlines the diagnosis, management, and treatment sequelae of an individual with prostate cancer and a known *TBX5* T-box transcription factor mutation. 

## 2. Case Report 

**Figure 1:**
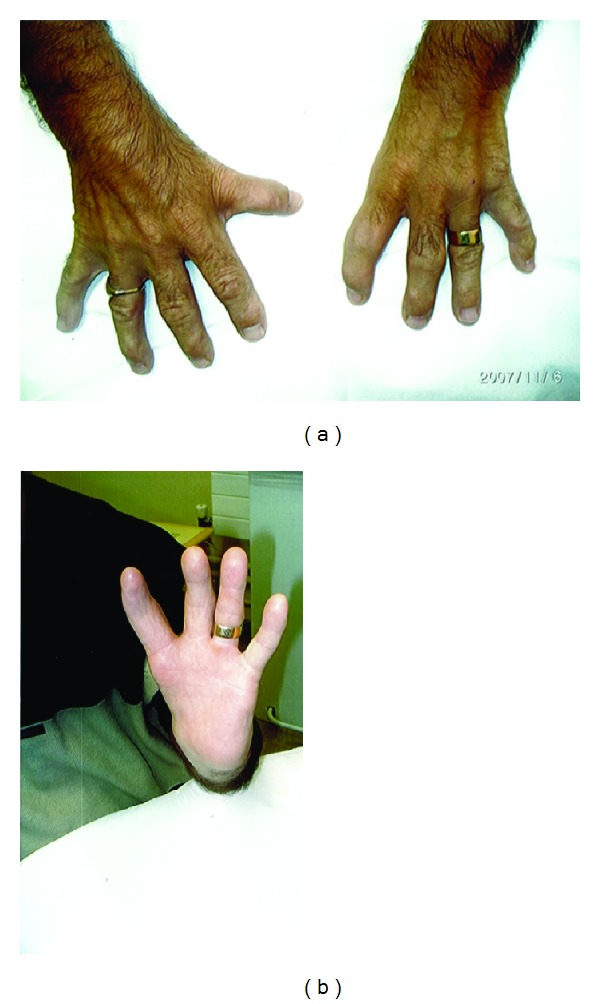
The patient's hands exhibit radial foreshortening, digit aplasia, and triphalangeal thumb of the right hand.

A 41-year-old male received three-dimensional conformal radiation therapy (3DCRT) as monotherapy for an early stage carcinoma of the prostate. He had an opportunistic prostate specific antigen (PSA) which was raised at 8.9 ng/mL and on staging workup was found to have a cT2aN0M0, Gleason score 3 + 3 prostate adenocarcinoma. He had a background of Holt-Oram syndrome (hand-heart syndrome), an autosomal dominant condition characterised by upper limb and cardiac defects [1]. The patient had bilateral radial foreshortening, triphalangeal thumbs, digit aplasia (Figure 1), and a cardiac dysrhythmia for which he had a pacemaker in situ. 

Following multidisciplinary assessment, he was elected to have 3DCRT which was well tolerated without any Grade 3 or 4 urinary or gastrointestinal toxicity, and he is in biochemical remission with an undetectable PSA five years after completion of treatment. He has no enhanced normal tissue toxicity. 

## 3. Discussion

Holt-Oram syndrome is caused by mutations in *TBX5* [2], a T-box transcription factor gene family member coded for by a gene on chromosome 12q2. There have been only two previously reported clinical cases of Holt-Oram syndrome associated with malignancy to date [3, 4], with parotid carcinoma and lymphosarcoma, respectively. However, it is now known that other T-box transcription factors can act as tumour suppressor genes. Another T-box factor, *TBX3, *can cause absent or hypoplastic mammary glands and it is under investigation in breast cancer carcinogenesis. The *TBX2* gene is deregulated in melanoma, breast, and pancreatic cancers where it suppresses senescence through the repression of cyclin-dependent kinase inhibitors, p19 and p21. Although this is the first case of prostate cancer reported in a patient with the *TBX5* mutation, it has been demonstrated that T-box transcription factors have an important role in metastasis in murine models of prostate carcinoma [5]. 

In conclusion, our case demonstrates the importance of screening patients with rare genetic disorders for cancer and the growing knowledge of the genetic basis of prostate cancer.
